# Identification of hub biomarkers and immune cell infiltration characteristics of polymyositis by bioinformatics analysis

**DOI:** 10.3389/fimmu.2022.1002500

**Published:** 2022-09-26

**Authors:** Qi Jia, Rui-Jin-Lin Hao, Xiao-Jian Lu, Shu-Qing Sun, Jun-Jie Shao, Xing Su, Qing-Feng Huang

**Affiliations:** ^1^ Department of Neurosurgery, Affiliated Hospital of Nantong University, Nantong, China; ^2^ Medical School of Nantong University, Nantong, China; ^3^ Department of Anesthesiology, Affiliated Hospital of Nantong University, Nantong, China

**Keywords:** polymyositis, biomarker, LASSO algorithm, weighted gene co-expression network analysis, immune cell infiltration, single-sample gene set enrichment analysis

## Abstract

**Background:**

Polymyositis (PM) is an acquirable muscle disease with proximal muscle involvement of the extremities as the main manifestation; it is a category of idiopathic inflammatory myopathy. This study aimed to identify the key biomarkers of PM, while elucidating PM-associated immune cell infiltration and immune-related pathways.

**Methods:**

The gene microarray data related to PM were downloaded from the Gene Expression Omnibus database. The analyses using Gene Ontology (GO), Kyoto Encyclopedia of Genes and Genomes, gene set enrichment analysis (GSEA), and protein-protein interaction (PPI) networks were performed on differentially expressed genes (DEGs). The hub genes of PM were identified using weighted gene co-expression network analysis (WGCNA) and least absolute shrinkage and selection operator (LASSO) algorithm, and the diagnostic accuracy of hub markers for PM was assessed using the receiver operating characteristic curve. In addition, the level of infiltration of 28 immune cells in PM and their interrelationship with hub genes were analyzed using single-sample GSEA.

**Results:**

A total of 420 DEGs were identified. The biological functions and signaling pathways closely associated with PM were inflammatory and immune processes. A series of four expression modules were obtained by WGCNA analysis, with the turquoise module having the highest correlation with PM; 196 crossover genes were obtained by combining DEGs. Subsequently, six hub genes were finally identified as the potential biomarkers of PM using LASSO algorithm and validation set verification analysis. In the immune cell infiltration analysis, the infiltration of T lymphocytes and subpopulations, dendritic cells, macrophages, and natural killer cells was more significant in the PM.

**Conclusion:**

We identified the hub genes closely related to PM using WGCNA combined with LASSO algorithm, which helped clarify the molecular mechanism of PM development and might have great significance for finding new immunotherapeutic targets, and disease prevention and treatment.

## Introduction

Polymyositis (PM), which is a category of idiopathic inflammatory myopathy, is a heterogeneous autoimmune disease that mainly involves skeletal muscle and presents with symmetrical weakness of the proximal muscle groups. Besides the skeletal muscle manifestations, other organ systems, including the heart, gastrointestinal tract, and lungs, may also be involved (1, 2). At the same time, PM may also increase the risk of malignancy (3), and its common complications and causes of death are interstitial lung disease and malignancy (3, 4). The prevalence of PM and dermatomyositis (DM) was estimated to be (5-21.5)/100,000 people, with two peaks in the age of onset, 10-15 years and 45-60 years (5, 6). The exact pathogenesis of PM is still unclear. Its development may be the result of multiple factors, such as infections, immune abnormalities, genetic factors, and environmental factors (7, 8). However, increasing clinical and experimental evidence suggests that the immune cells and immune-related pathways play an essential role in PM development. It is generally accepted that the typical pathological change in PM is an increase in the number of CD8+ T cells in the endomysial area around the muscle fibers, forming a “CD8+/Major Histocompatibility Complex-1(MHC-1) complex.” The complex releases perforin granules, leading to muscle lysis and destruction (9, 10). Moreover, the infiltration of CD4+ T lymphocytes, macrophages, and dendritic cells and the release of various pro-inflammatory cytokines, such as interleukin 1, interleukin 6, IL-15, interleukin-18, tumor necrosis factor-a, and type I interferons (IFNs), were found in PM muscle tissue (11–13). However, the exact molecular mechanisms of action need to be further clarified. Glucocorticoids and immunosuppressants are the main treatments for PM/DM, but some patients still have poor outcomes. Hence, it is of great significance to further investigate the pathogenesis of PM and find new therapeutic targets for preventing and treating the disease.

**Figure 1:**
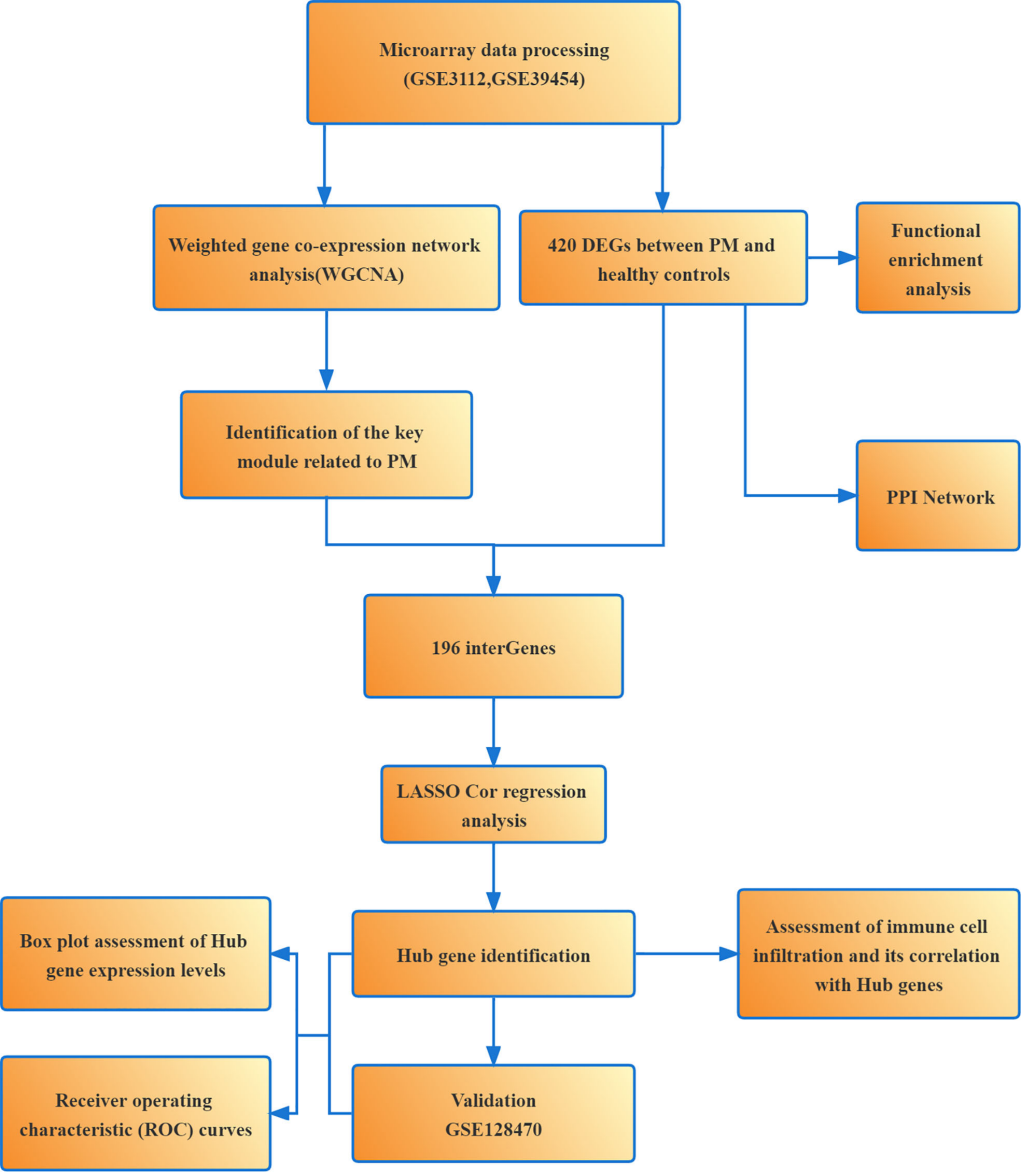
Flowchart of the present study.

Weighted gene co-expression network analysis (WGCNA) and LASSO are commonly used methods for bioinformatics analysis. WGCNA is a systems biology approach to describe the genetic association patterns between different samples. It can be used to identify highly synergistic genomes and determine the candidate biomarker genes or therapeutic targets based on the intrinsic associations between genomes and phenotypes (14). LASSO algorithm is characterized by variable selection and regularization while fitting a generalized linear model, thus specifying the exact degree of association between the two variables of interest (15). LASSO analysis of the resulting modular signature genes from WGCNA can improve the accuracy of screening for biomarker genes or therapeutic targets. Subsequently, we performed enrichment analysis of the differentially expressed genes (DEGs) to understand their associated biological functions and signaling pathways. We also analyzed the infiltration of 28 immune cells in PM samples using single-sample gene set enrichment analysis (ssGSEA) to clarify the correlation between immune cell infiltration and gene expression, which might help elucidate the pathogenesis of PM and developing new immunotherapeutic targets (**Figure 1**).

## Materials and methods

### Microarray data download and pre-processing

Three PM-related datasets were obtained from the Gene Expression Omnibus database (http://wwwncbinlmnihgov/geo). The specific information is provided in **Table 1**. GSE3112 and GSE128470 sequencing platforms were both GPL96 (Affymetrix Human Genome U133A Array). The GSE39454 sequencing platform was GPL570 (Affymetrix Human Genome U133 Plus 2.0 Array). The probes were converted into the corresponding gene symbols according to the annotation information of the respective platforms of the dataset. The “SVA” package was applied to eliminate the batch effect between the GSE3112 and GSE39454 microarray expression matrices, and the two were combined as the training dataset. The GSE128470 microarray expression matrix was used as a separate validation dataset.

**Table 1:** The basic information of the databases.

Database ID	Platform	Author	Year	Tissue sample	Number of samples	Number of controls
**Training set**						
GSE3112	GPL96	Steven Alan Greenberg	2005	Skeletal muscle	6 polymyositis samples	11 healthy controls
GSE39454	GPL570	Wei Zhu	2012	Skeletal muscle	8 polymyositis samples	5 healthy controls
**Validation set**						
GSE128470	GPL96	Steven A Greenberg	2019	Muscle	7 polymyositis samples	12 healthy controls

### Screening of DEGs

Differentially expressed genes (DEGs) were screened using the “Limma, ggplot2” package for the training dataset with an adjusted P value <0.05 and |log fold change (FC)|>1.

### Functional enrichment analysis and construction of the protein–protein interaction network

Using the “clusterProfiler, enrichplot” package, DEGs were enriched for Gene Ontology (GO) and Kyoto Encyclopedia of Genes and Genomes (KEGG) based on the screening criteria of P <0.05 and adjusted P value <0.05. The immune gene sets from the Molecular Signatures Database (MsigDB) (http://softwarebroadinstituteorg/gsea/msigdb) were used as a reference for GSEA analysis, where |NES| ≥1, P <0.05, and false discovery rate q values <0.05 were taken as significant for enrichment. The protein-protein interaction (PPI) networks for DEGs were subsequently constructed using STRING (http://string-dborg) with a minimum interaction score of ≥0.9.

### Construction of gene co-expression network

WGCNA is a systems biology approach to find gene modules highly correlated with clinical phenotypes. It is commonly used to identify and screen for disease markers in organisms. The WGCNA program package in R software was used to construct a weighted co-expression network on the training set. First, the “goodSamplesGenes” function was used to find the missing values and organize the data set. Then, a desirable soft threshold (β) was selected under the validation of the “pickSoftThreshold” function. The disordered neighborhood relationships between genes were truncated based on the soft threshold size, followed by transforming the matrix data into a neighborhood matrix and building a scale-free topological network. The adjacency matrix was transformed into a topological overlap matrix based on the topological overlap differences in network connection strength. Subsequently, we set the restricted minimum number of genes per module to 60 and used a threshold of 0.25 to merge similar modules, while also drawing a hierarchical clustering dendrogram, to perform gene clustering and dynamic module identification. We then plotted the heat maps of module-clinical trait correlations based on module eigengene. The gene significance (GS) and module membership (MM) were also calculated to assess the significance of genes and the correlation between genes and modules.

### Identification and validation of hub genes

The absolute value of GS >0.50 and the absolute value of MM >0.80 were used as the screening criteria to select the gene module of the highest GS value as the candidate hub gene. The candidate hub genes were intersected with DEGs using the “venn” package of R software, and then the intersected genes were subjected to LASSO analysis using the “glmnet” package to filter the hub genes. Box plots were constructed using the “ggpubr” package to evaluate the expression levels of the hub genes in PM and healthy controls. The receiver operating characteristic (ROC) curves were plotted using the “pROC” package to determine the accuracy of hub genes as diagnostic genes. Then, GSE128470, an independent external dataset, was used as a validation dataset to evaluate the expression level and diagnostic value of the pivotal genes. The final hub genes were derived after excluding genes with large errors.

### Assessment of immune cell infiltration and its correlation with hub genes

The relative infiltration levels of 28 immune cells in PM were analyzed using the ssGSEA algorithm. The differences in immune cells between PM and healthy control groups were analyzed using the “vioplot” package. Spearman correlation analysis was then applied to calculate the association between the hub genes and the 28 immune infiltrating cells as well as to visualize the results.

## Results

### Screening of DEGs

**Figure 2:**
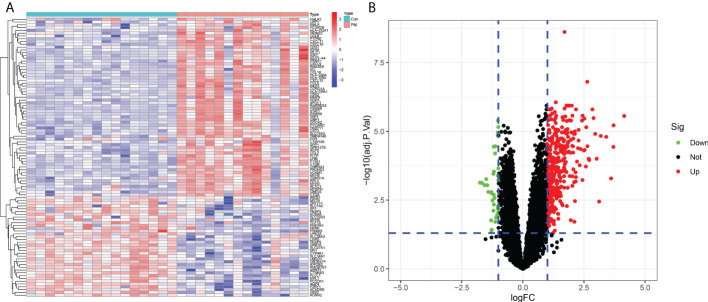
Differentially expressed genes between patients with polymyositis (PM) and healthy controls. **(A)** Heatmap of the top 50 upregulated and downregulated genes. **(B)** Volcano plot for DEGs between healthy controls and PM tissues.

We identified 420 DEGs in patients with PM, including 384 upregulated genes and 36 downregulated genes at adjusted P <0.05 and |logFC|>1, after merging and eliminating batch effects in the GSE3112 and GSE39454 data sets (**Figure 2**). Also, 12,548 overlapping genes were acquired for WGCNA analysis.

### Functional enrichment analysis and construction of the PPI network

**Figure 3:**
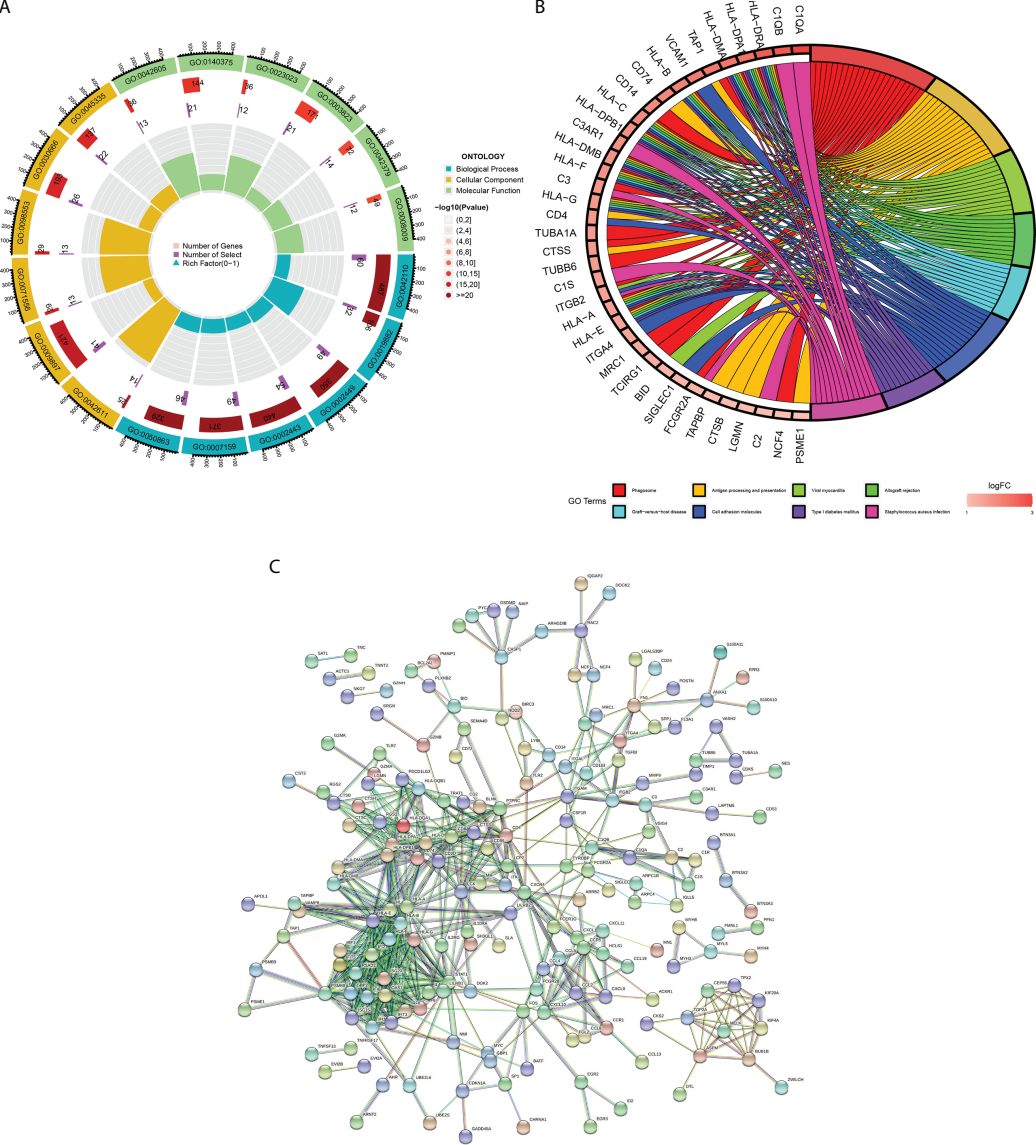
Functional enrichment analysis and PPI network of DEGs. **(A)** GO enrichment analysis. The first circle indicates the name of GO; the second circle represents the number of genes on each GO. (the redder the color, the more significant the DEGs enrichment); the third circle represents the number of differential genes enriched on each GO term; and the fourth circle represents the proportion of genes. **(B)** KEGG pathway enrichment analysis. The first eight pathways with significant enrichment of differential genes are demonstrated. **(C)** Protein-protein interaction (PPI) network.

**Figure 4:**
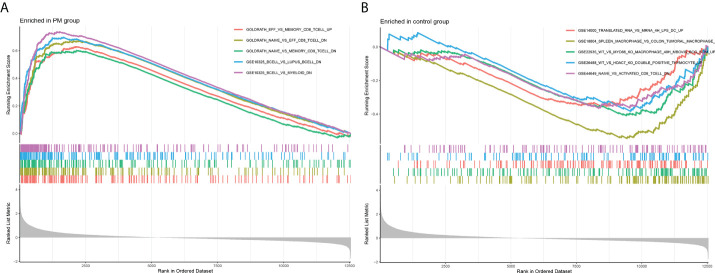
Enrichment plot for GSEA. **(A)** Active gene set in PM group. **(B)** Active gene sets in healthy controls.

We learned about the biological functions and signaling pathways closely related to PM through GO and KEGG enrichment analyses of DEGs. The biological processes mainly included inflammation and immune response–related processes, such as proliferation activation of immune cells (e.g., lymphocytes, dendritic cells, and macrophages), cytokine formation [e.g., type I interferon (type I IFN), IFN-γ, and IFN-β], complement activation, immune response, and immunomodulatory effects; defense response processes, such as defense response to viruses and defense response to symbionts; and biological membrane–related functions, e.g., plasma membrane signaling receptor complex and integral component of endoplasmic reticulum membrane (**Figure 3A**). The signaling pathway analysis included mainly inflammatory and immune-related diseases (e.g., Epstein-Barr virus infection and systemic lupus erythematosus) as well as immune response–related pathways [antigen processing and presentation, natural killer (NK) cell-mediated cytotoxicity, and T cell receptor signaling pathway] (**Figure 3B**). GSEA showed that the gene set was mainly enriched in macrophages, B cells, CD4+ T cells, CD8+ T cells, cytokines, and immune organs in the PM group (**Figures 4A, B**). **Table 2** shows the top five enriched gene sets in the PM group and healthy controls. We further visualized the gene information and constructed a PPI network of DEGs with a PPI enrichment P value <1.0e-16. The network consisted of 650 edges and 407 nodes with tight connections among the nodes (**Figure 3C**).

**Table 2:** Top 5 significantly enriched gene sets in PM group and healthy control group.

Gene set name	NES	pvalue	p.adjust	qvalues
Enriched in PM group				
GOLDRATH Eff vs memory CD8 T cell up	2.398	1.00E-10	1.51E-09	7.60E-10
GOLDRATH Naive vs eff CD8 T cell down	2.549	1.00E-10	1.51E-09	7.60E-10
GOLDRATH Naive vs memory CD8 T cell down	2.282	1.00E-10	1.51E-09	7.60E-10
GSE10325 B cell vs lupus B cell down	2.665	1.00E-10	1.51E-09	7.60E-10
GSE10325 B cell vs myeloid down	2.823	1.00E-10	1.51E-09	7.60E-10
Enriched in control group				
GSE14000 Translated RNA vs mRNA 4h lps dc up	-1.647	< 0.001	< 0.001	< 0.001
GSE18804 Spleen macrophage vs colon tumoral macrophage down	-2.526	1.00E-10	1.51E-09	7.60E-10
GSE22935 WT vs myd88 ko macrophage 48h mbovis bcg stim up	-1.894	7.30E-07	5.24E-06	2.64E-06
GSE26488 WT vs hdac7 ko double positive thymocyte up	-1.742	< 0.001	< 0.001	< 0.001
GSE44649 Naive vs activated CD8 T cell down	-1.675	< 0.001	0.001	< 0.001

### Construction of weight gene co-expression network and identification of the key module

**Figure 5:**
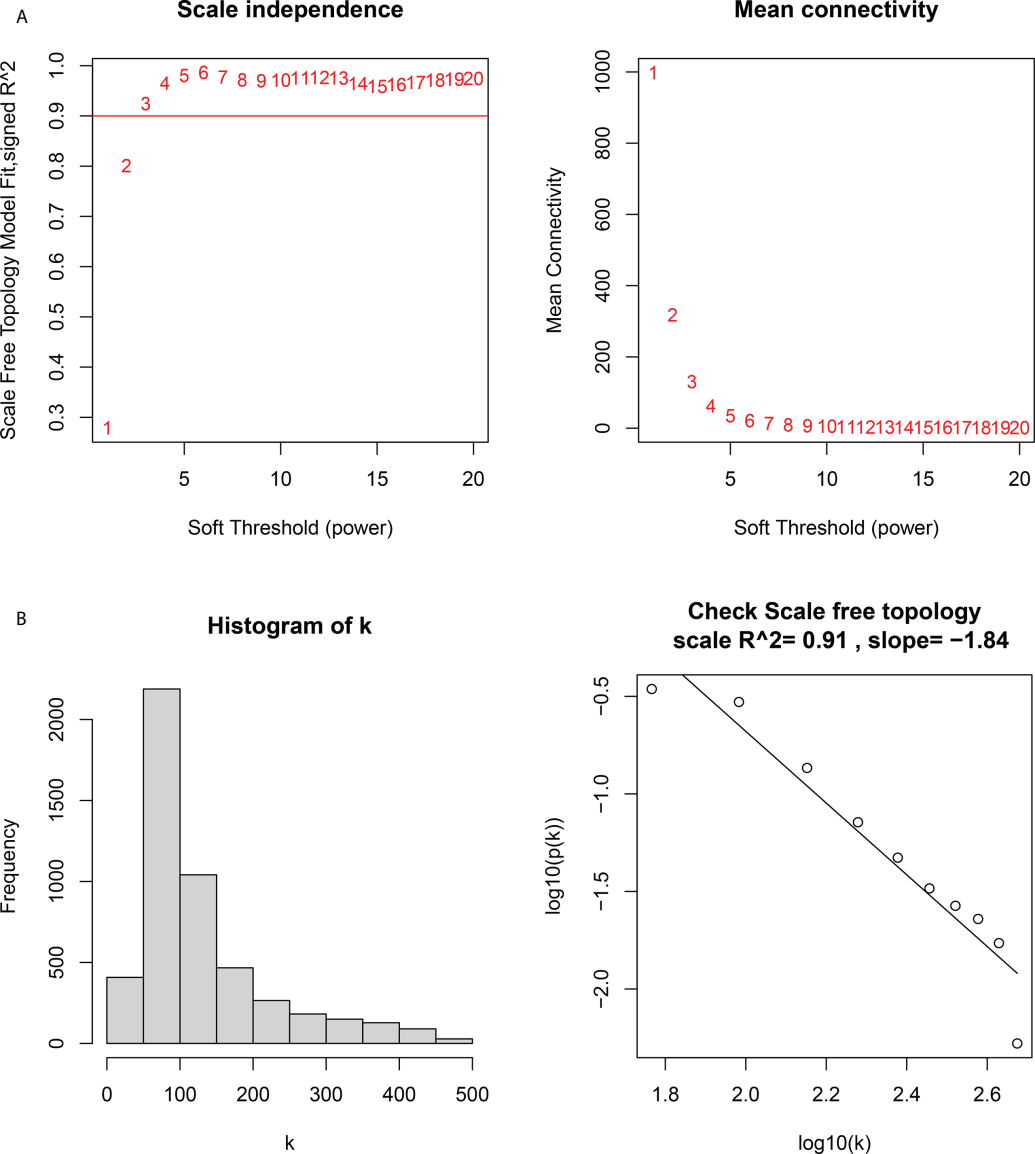
Determination of the soft threshold power in the weighted gene co-expression network analysis (WGCNA). **(A)** Left: analysis of the scale-free fit index for various soft threshold powers. Right: analysis of the mean connectivity for various soft threshold powers. **(B)** Histogram of connectivity distribution and checking the scale-free topology.

**Figure 6:**
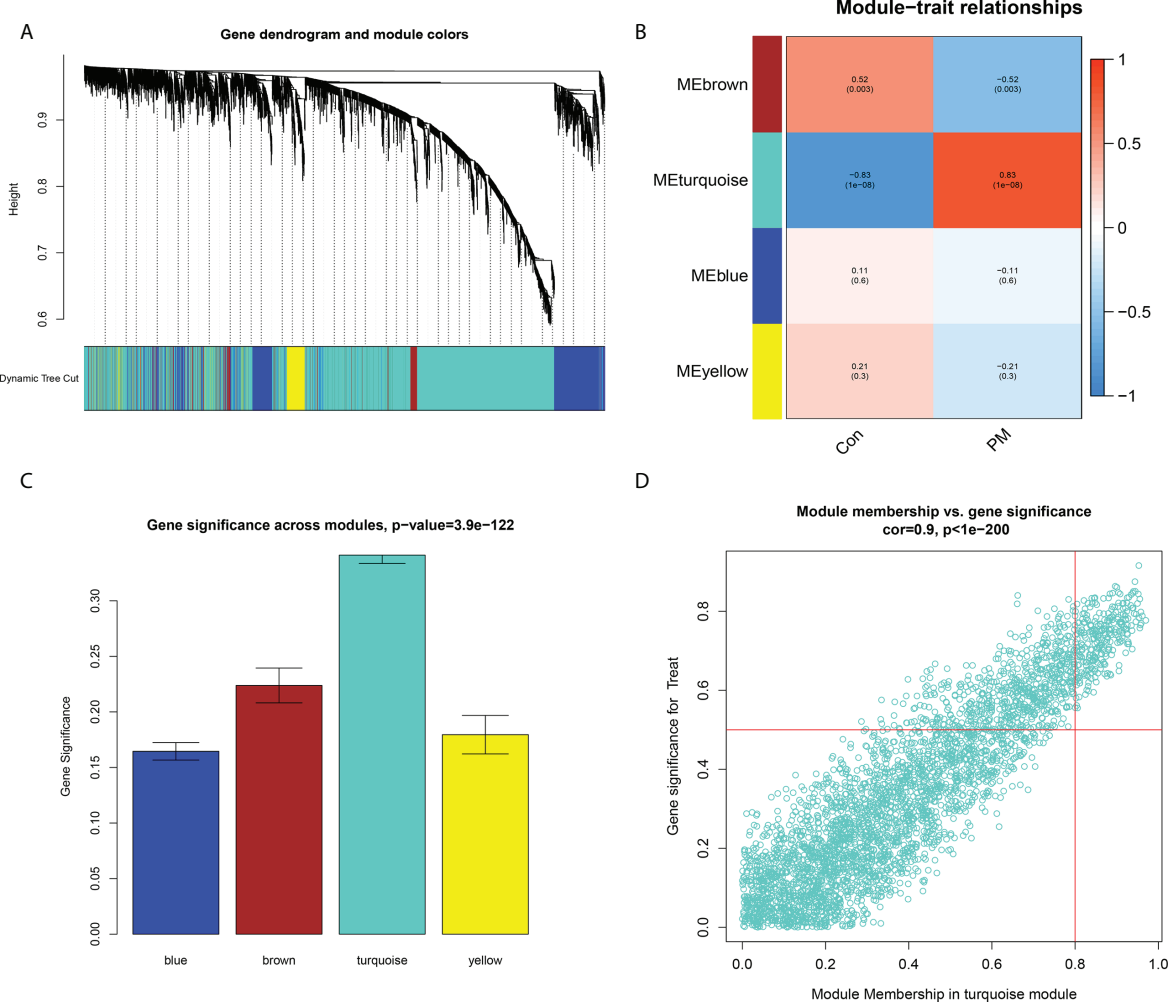
Construction of Weight Gene Co-Expression Network and Identification of the Key Module. **(A)** Clustering dendrogram of genes. In the figure, each branch represents a gene and on the bottom each color represents a co-expression module. **(B)** Heatmap of the association between module genes and clinical traits. **(C)** Gene significance in the modules. **(D)** Module membership and gene significance analyses of the turquoise module.

We constructed a WGCNA network using the 12,548 overlapping genes obtained from the difference analysis to identify the hub genes in PM development. The samples were first screened to remove missing values to ensure the reliability of the network construction, and a soft threshold β = 3 (scale-free R2 = 0.91; slope = –1.84) was determined (**Figure 5**). Then, the co-expression matrix was constructed. We obtained four gene modules by dynamic shearing module identification and similar module merging (**Figure 6A**). Using module–clinical trait correlation heat map analysis, we found that the turquoise color module was the gene module with the highest correlation with PM (cor = 0.83, P = 1e-08) (**Figures 6B, C**). Subsequently, 286 core genes of the turquoise module (cor = 0.9, P < 1e-200) were screened according to GS >0.5 and MM >0.8 (**Figure 6D**) for subsequent analysis.

### LASSO analysis and validation of hub genes

**Figure 7:**
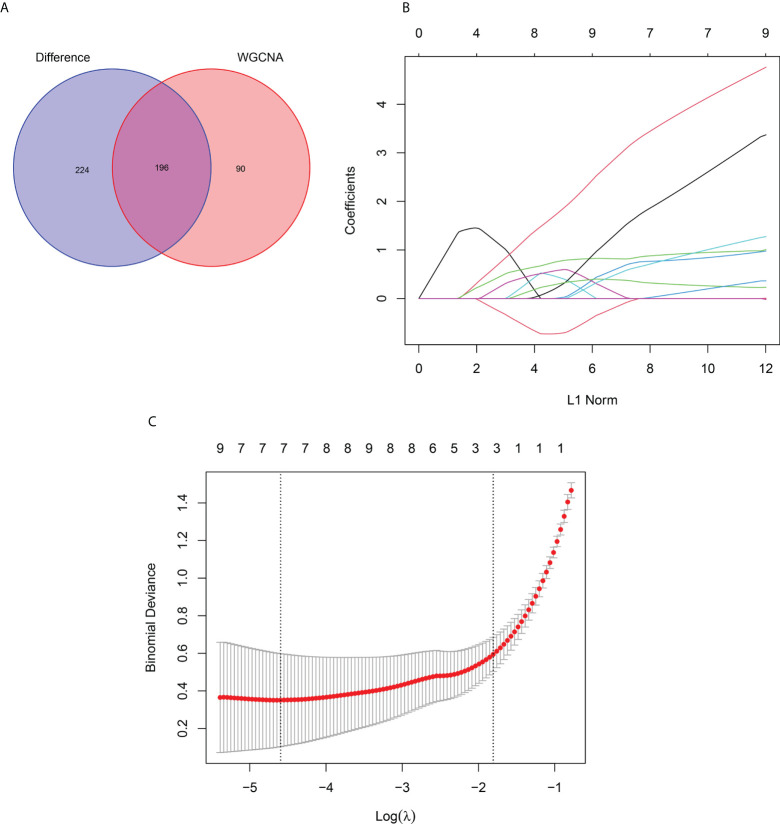
LASSO Analysis and Validation of Hub Genes. **(A)** Venn diagram of intersecting genes between DEGs and the turquoise module. **(B)** Coefficients distribution trend of LASSO regression. **(C)** Distribution of hub genes in cross validation.

First, the DEGs were crossed with the key gene module to obtain 196 intersecting genes (**Figure 7A**). Subsequently, seven genes were screened as the hub genes of PM from the intersecting genes using LASSO analysis, including ANXA1, BID, CECR1, CHN1, MARCKSL1, migration inhibitory factor (MIF), and SSR4 (**Figures 7B, C**).

### Identification and validation of hub gene expression levels and diagnostic value

**Figure 8:**
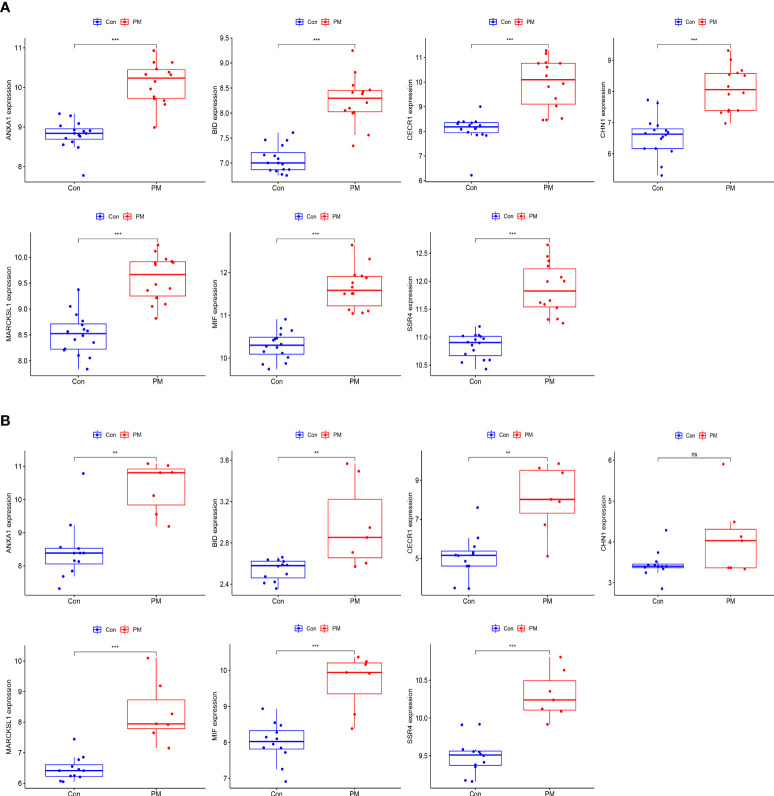
Expression levels of hub genes. Boxplot of hub genes in training dataset **(A)** and validation dataset **(B)**. (***p<0.001; **p<0.01; *p<0.05; ns, no significance).

**Figure 9:**
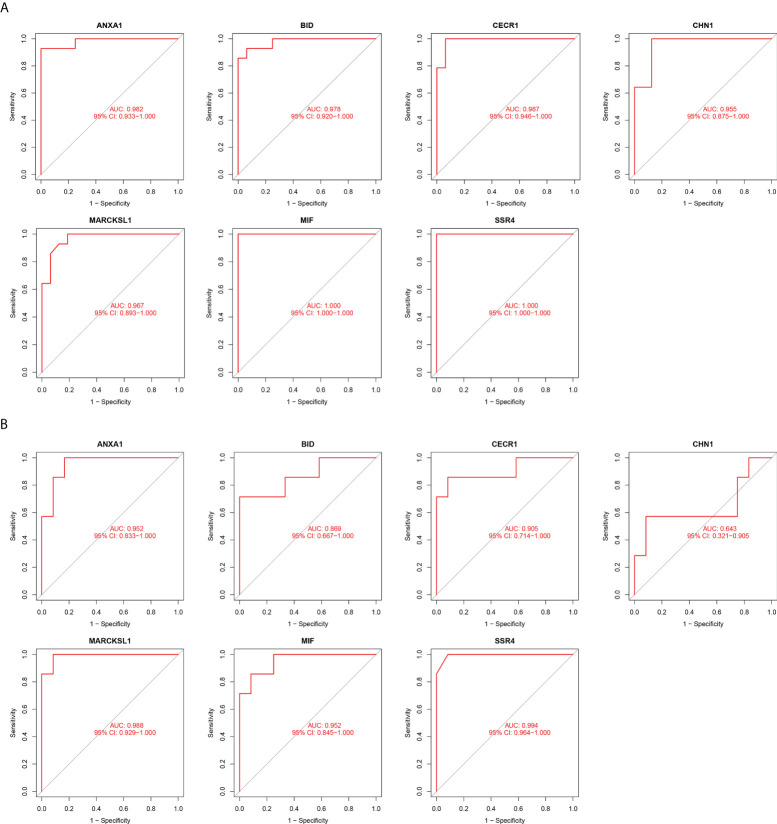
Diagnostic Value of hub genes. ROC curves of hub genes in the training dataset **(A)** and validation dataset **(B)**. (Higher the value of AUC, better the diagnostic value of the gene.).

We used box plots to determine the expression levels of seven hub genes in the PM group and healthy controls. The results showed that ANXA1, BID, CECR1, CHN1, MARCKSL1, MIF, and SSR4 were all expressed at significantly higher levels in the PM group than in the healthy control group (P < 0.001) (**Figure 8A**). Subsequently, the accuracy and specificity of the seven hub genes as the disease diagnostic genes were determined using the area under the ROC curve (AUC). The results showed that the AUC values of the seven hub genes were >0.95 (**Figure 9A**). This indicated that all the hub genes obtained from our screening had a high diagnostic value. Then, we used the independent dataset GSE128470 as the validation dataset to identify its expression level and diagnostic value so as to further validate the clinical application value of the hub genes. The results suggested that MARCKSL1, MIF, and SSR4 were expressed at significantly higher levels in the PM group than in the healthy control group in the validation set (P < 0.001), whereas ANXA1, BID, and CECR1 had higher expression levels in the PM group (P < 0.01), but the differential expression of CHN1 in the PM and healthy control groups was not statistically significant (**Figure 8B**). The ROC curve was used to further validate the diagnostic value of the seven hub genes in the validation group. The results showed that ANXA1, MARCKSL1, MIF, and SSR4 had a high diagnostic value with the AUC value >0.95; BID and CECR1 had a high diagnostic value with the AUC value >0.85; but CHN1 had a low diagnostic value with the AUC value = 0.643 (**Figure 9B**). After validation, the error of the CHN1 gene as the hub gene of PM was high, and hence we decided to exclude it. ANXA1, BID, CECR1, MARCKSL1, MIF, and SSR4 were used as the final hub genes of PM for subsequent analysis.

### Assessment of immune cell infiltration and its correlation with hub genes

**Figure 10:**
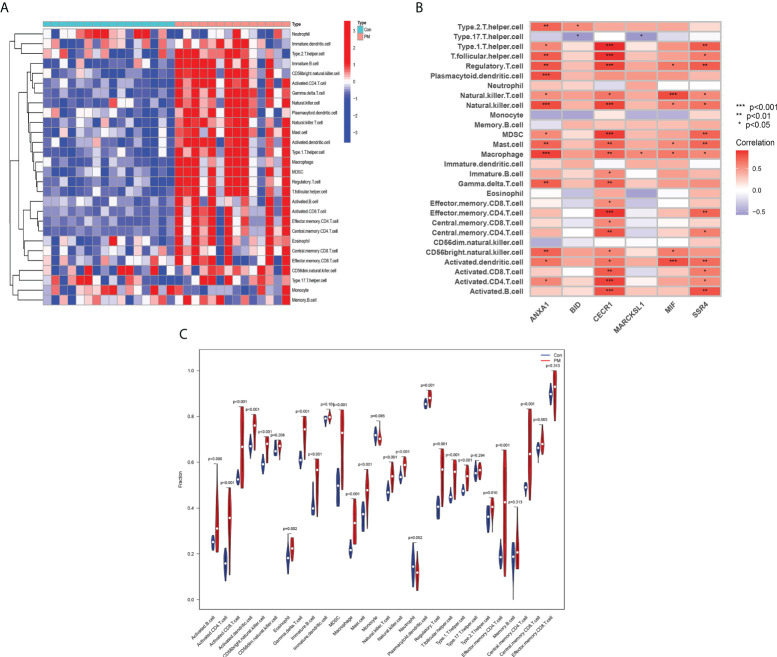
Assessment of Immune Cell Infiltration and Its Correlation With Hub Genes. Heat map **(A)** and Violin diagram **(B)** of immune cell infiltration. **(C)** Relationship between immune cell infiltration and six hub genes.

The relative infiltration levels of 28 immune cells in the PM group compared with healthy controls were obtained by ssGSEA algorithm analysis, as shown in **Figure 10A**. The infiltration of macrophages, eosinophils, mast cells, dendritic cells, activated B cells, NK cells, T lymphocytes, and subpopulations was more remarkable in the PM group in the immune cell differential analysis (**Figure 10C**). We performed a correlation analysis to further explore the association of hub genes with 28 immune cells.

ANXA1 was positively correlated with macrophages, NK cells, and plasmacytoid dendritic cells at P <0.001; type 2 T helper cells, T follicular helper cells, regulatory T cells, mast cells, γ-δ-T cells, and CD56bright NK cells at P <0.01; and type 1 T helper cells, NK T cells, and activated CD4 T cells at P <0.05. BID was positively correlated with type 2 T helper cells (P < 0.05) and negatively correlated with type 17 T helper cells (P < 0.05). CECR1 was positively correlated with type 1 T helper cells, T follicular helper cells, regulatory T cells, NK cells, Myeloid-derived suppressor cells (MDSCs), effector memory CD4 T cells, and activated B cells at P <0.001; mast cells, macrophages, γ-δ-T cells, central memory CD4 T cells, and activated CD8 T cells at P <0.01; NK T cells, immature B cells, effector memory CD8 T cells, central memory CD8 T cells, CD56bright natural killer cells, and activated dendritic cells at P <0.05. MARCKSL1 was positively correlated with macrophages (P <0.05) and negatively correlated with type 17 T helper cells (P <0.05). MIF was positively correlated with NK T cells and activated dendritic cells at P <0.001; regulatory T cells, NK cells, macrophages, mast cells, and CD56bright NK cells. SSR4 was positively correlated for type 1 T helper cells, regulatory T cells, MDSC, mast cells, effector memory CD4 T cells, activated dendritic cells, and activated B cells at P <0.01, and for T follicular helper cells, NK cells, NK T cells, macrophages, central memory CD4 T cells, activated CD8 T cells, and activated CD4 T cells at P <0.05. The aforementioned findings indicated that the hub genes might play an important role in the PM immune response (**Figure 10B**).

## Discussion

PM is an autoimmune disease with skeletal muscle involvement as the main manifestation and is a rare clinical condition. Unlike dermatomyositis, PM does not have the same early rash-like changes and the actual beginning of the disease is not easily determined. The onset of PM is insidious, and the disease may be delayed for a long time, leading to typical symptoms only over months or years. The diagnosis cannot be easily confirmed in clinical work and is very often used as an exclusionary diagnosis, which is a frequently misdiagnosed disease (5, 16, 17). Therefore, the clarification of the key biomarkers of PM is of great value for the early diagnosis and treatment of the disease.

We obtained 420 DEGs, and the biological functions closely related to these DEGs included the proliferation activation of immune cells such as T cells, B cells, and macrophages; the formation of cytokines such as type I IFN and tumor necrosis factor alpha; the activation of the complement; and the defense response processes such as defense response to viruses. Some researchers found the infiltration of T cells, macrophages, and dendritic cells in the muscle tissue of patients with PM using immunohistochemical methods for phenotyping inflammatory cells in muscle biopsies (18), as well as the presence of various cytokines (e.g., type I IFN and IL-6) and autoantibodies in muscle tissue (2, 8, 11, 19, 20). It has been found that patients with PM/DM have a significant type I IFN phenotype in muscle fibers as well as in peripheral blood (21). In addition, the serum containing anti-Jo-1 autoantibodies was extracted from patients with PM and found to have the ability to induce IFN production when bound to necrotic cells *in vitro* (8, 22, 23). Therefore, the targeted modulation of type I IFN is a promising therapeutic approach for PM that can be further explored in patients with anti-Jo-1 antibodies or in patients with an IFN phenotype. In addition, it has also been demonstrated that serum IL-6 levels correlate with disease activity in patients with DM and PM (24). A recent study found that CD28null T cells were associated with disease activity in patients with myositis (25). A high percentage of CD28null T cells persisted in the muscle tissue of patients with myositis after glucocorticoid treatment. Meanwhile, CD28null T cells had a higher degree of myotoxicity against autologous myotubes compared with conventional T cells (8). CD28null T cells are thus a new target for treating patients with refractory PM/DM. All these studies supported the idea that the inflammatory and immune mechanisms were involved in the pathogenesis of PM. The analysis of the KEGG signaling pathway revealed that the DEGs were mainly associated with the bracketed inflammatory and immune-related diseases and immune response–related pathways such as chemokine signaling pathway, T cell receptor signaling pathway, Toll-like receptor signaling pathway, and Tumor necrosis factor (TNF) signaling pathway. In the muscle tissue of patients with PM, CD8+ T cell-induced signaling pathways were characteristic processes of disease progression. TCR-mediated cascade responses might be a key pathway in determining circulating T cell responses associated with disease activity in PM/DM (26). GSEA showed that the gene set was mainly enriched in macrophages, B cells, CD4+ T cells, CD8+ T cells, cytokines, immune organs, and so forth, in the PM group. This further indicated that the development of PM was closely related to inflammatory and immune processes.

Screening for disease signature genes by combining WGCNA and LASSO algorithm avoids the limitations of traditional study methods and allows for a more comprehensive exploration of the disease. We identified seven genes as hub genes, including ANXA1, BID, CECR1, CHN1, MARCKSL1, MIF, and SSR4. Then, we used GSE128470 as a validation dataset to identify the expression level and diagnostic value of the hub genes, removing genes with large errors. Finally, ANXA1, BID, CECR1, MARCKSL1, MIF, and SSR4 were identified as the hub genes of PM. Macrophage migration inhibitory factor (MIF) is a pleiotropic cytokine secreted by activated T cells and macrophages that prevents the random migration of macrophages. Recent studies have identified MIF as a pro-inflammatory cytokine (27–30) that plays an essential role in inflammatory and autoimmune diseases. MIF induces IL-6 and tumor necrosis factor-α production, which are considered to be the cytokines closely associated with disease activity in patients with PM (9). An enzyme-linked immunosorbent assay of MIF in the serum from patients with PM patients showed that the MIF levels were significantly higher in the serum from patients with active PM than in the serum from patients in remission and healthy individuals (31). An experiment exploring the amount and distribution of MIF in inflammatory myopathies revealed that the MIF levels were markedly greater in the PM group than in the control group in muscle samples; however, no significant differences were observed in other inflammatory diseases (32). Therefore, MIF may be an important biomarker of PM activity and prognosis. Lately, it has been found that MIF exerts antagonistic effects on glucocorticoids *in vitro* and *in vivo*. Recombinant MIF antagonizes glucocorticoid-induced inhibition of cytokine production by macrophages and T lymphocytes. MIF inhibits the therapeutic effects of glucocorticoids in mice with antigen-induced arthritis (29, 33–35). However, paradoxically, no significant difference has been found in the serum MIF concentration between patients with active PM treated and not treated with glucocorticoids; also, no relationship has been noted between serum MIF concentration and glucocorticoid dose (31). Therefore, the relationship between MIF and glucocorticoids in patients with PM needs to be further investigated. ANXA1, BID, CECR1, MARCKSL1, and SSR4 have not been reported to be associated with PM, but these genes are closely associated with immune inflammatory responses or tumors and may be linked to the development of PM and the mechanism of combined malignancy. Annexin A1 (ANXA1) is a 37-kDa calcium-dependent phospholipid-binding protein known as a membrane-linked protein. The homologous receptor for ANXA1 is the formylated peptide receptor (FPR). It is a G protein-coupled receptor with seven transmembrane regions, which is expressed mainly by mammalian phagocytes and plays an important role in defense and inflammation (36). The activation of this pathway produces different results in different cell types. For example, the activation of the pathway leads to the differentiation of monocytes into macrophages and dendritic cells as well as the induction of T cell proliferation, but it also leads to the apoptosis and necrosis of neutrophils (37, 38). The ANXA1/FPR signaling pathway also plays an important role in acquired immunity. A previous study found the high expression of anti-ANXA1 autoantibodies in patients with ulcerative colitis and Crohn’s disease and a direct correlation of the potency of the antibodies with disease activity. Due to the presence of both the anti-inflammatory protein ANXA1 and its autoantibodies during disease progression, it was hypothesized that ANXA1 and its derivatives might act as antigens to promote pathogenic autoantibody production in the immune inflammatory response (39, 40). This idea was further supported by a study in which the antibodies to ANXA1 were detected in the serum and bronchoalveolar lavage fluid of patients with acute exacerbations of idiopathic pulmonary fibrosis (IPF). It was also shown that ANXA1 functioned as an autoantigen to promote antibody production and T cell responses in patients with IPF during acute exacerbations, thereby exacerbating disease progression (41). Whether ANXA1 and its autoantibodies play a similar role in PM and PM-associated IPF needs to be determined by further studies. BID is a member of the pro-apoptotic Bcl-2 family containing only the BH3 structural domain. The related gene is localized on human chromosome 22q11 and is expressed in most tissues. After the cells are exposed to apoptosis-inducing factors (e.g., tumor necrosis factor, TNF-α) stimulated by apoptotic signals, BID is cleaved by activated caspase-8 to form a truncated BID fragment (tBID) in the inflammatory or tumor environment, which enters the mitochondria to induce the release of cytochrome c and other pro-apoptotic molecules to regulate apoptosis. It plays a major role in immune inflammatory response and tumor development (42–44). Adenosine deaminase gene (previously Cat Eye syndrome Chromosome Region, candidate 1) encodes the ADA2 protein. ADA2 is dimeric and secreted mainly by monocytes, myeloid cells, and lymphocytes. It has a low catalytic activity and functions only at high adenosine concentrations in environments such as hypoxia, inflammation, and tumors (45, 46). Studies have found a dramatic increase in ADA2 activity in immune diseases such as systemic lupus erythematosus and tuberculosis, while ADA2 levels in pleural effusions have also been used as a marker for tuberculosis (47, 48). In a case of ADA2 deficiency due to a mutation in the CECR1 gene, the patient presented with intermittent fever, cutaneous vasculitis, muscle pain, and muscle inflammation (49). This was similar to the manifestation of patients with PM involving skeletal muscle, suggesting that this enzyme could work as a biomarker to regulate immune responses in inflammation and cancer. The myristoylated alanine-rich C-kinase substrate (MARCKS) family includes two members, MARCKS and MARCKS-like protein 1 (MARCKSL1). Initially MARCKSL1 was also known as MacMARCKS because of its high expression in macrophages (50, 51). In fact, MARCKS and MARCKSL1 are closely associated with normal macrophage function, and both can be involved in macrophage migration through protein kinase C (PKC) or calcium-dependent phosphorylation of calmodulin binding, actin binding, and cytoplasmic translocation processes (52). Also, MARCKSL1 is involved in activities such as pro-intestinal formation, myogenesis, brain development and other developmental processes, cell adhesion regulation, cell phagocytosis, cell proliferation and differentiation, and angiogenesis (52–54). Signal sequence receptor subunit delta (SSR4) is a subunit of the translocon-associated protein complex and is present in the endoplasmic reticulum to direct the secretion and transport of immunoglobulins in the regulation of humoral immunity (55).

Our study was novel in analyzing the infiltration of 28 immune cell types in PM tissue using the ssGSEA algorithm. We found a more significant infiltration of T lymphocytes and subpopulations, dendritic cells, macrophages, and NK cells. T lymphocytes and subpopulations play a dominant role in PM muscle tissue, manifesting cytotoxic effects mediated by CD8+ T cells on the major histocompatibility complex class I (MHC class I) expressed by muscle fibers (9, 10). T helper (Th) cells amplify the immune response by secreting various cytokines and assist in the induction of antibody production through co-stimulatory signals (8, 56). Regulatory cells (Tregs) can inhibit the lytic activity of myoreactive CD8+ cells and are thought to be useful in balancing muscle inflammation (57). In addition, specific subtypes play a role in the progression of the autoimmune response, such as CD28null T cells and highly differentiated cytotoxic T cells (58). Also, a large infiltration of macrophages as well as dendritic cells was found in the muscle tissue of patients with PM, both of which produced large amounts of IL-18 in the endomysium of these patients. At the same time, high expression of IL-18R was detected in endothelial cells, smooth muscle cells, and CD8+ T cells, suggesting that the disruption of the IL-18/IL-18R pathway might be interrelated with the pathogenesis of PM. Therefore, it was hypothesized that CD8+ T cells could bind to IL-18 through overexpressed IL-8R, thus inducing cytotoxic effects, fibrous damage, and spread of inflammation. Following this, the detection of IL-18 expression levels could predict PM activity (59, 60).

In summary, we screened six hub genes associated with PM using a combination of WGCNA and LASSO: ANXA1, BID, CECR1, MARCKSL1, MIF, and SSR4. Meanwhile, the interconnection of these hub genes with PM was further described based on the present findings. Furthermore, a preliminary exploration of PM-associated immune cell infiltration and its relationship with hub genes was performed using the ssGSEA approach. Follow-up experiments with large samples are still needed to validate and analyze the value of hub genes as highly sensitive and specific diagnostic markers for PM. Meanwhile, a large number of molecular experiments are needed to explore the infiltration of various types of immune cells and their interrelationship with diagnostic markers, so as to further elucidate the mechanism of PM development, which is of great significance for the early clarification of PM diagnosis and targeted drug therapy.

## Data availability statement

Publicly available datasets were analyzed in this study. This data can be found here: https://www.ncbi.nlm.nih.gov/geo/query/acc.cgi?acc=GSE3112 https://www.ncbi.nlm.nih.gov/geo/query/acc.cgi?acc=GSE39454 https://www.ncbi.nlm.nih.gov/geo/query/acc.cgi?acc=GSE128470.

## Author contributions

Q-FH and XS conceived and designed the experimental procedure. QJ and R-J-LH performed the experiments and analyzed the data. QJ and Q-FH wrote the manuscript. Q-FH, QJ, XS, R-J-LH and X-JL revised the manuscript. S-QS and J-JS organized the images. All authors contributed to the article and approved the submitted version.

## Funding

The present study was supported by the Natural Science Foundation of Jiangsu Province (BK20130386), the National Natural Science Foundation of China (81402447), the Jiangsu Planned Projects for Postdoctoral Research Funds (no. 1402200C).

## Acknowledgments

We sincerely acknowledge the GEO database for providing online resources for gene expression and the researchers for uploading their meaningful datasets. We also sincerely appreciate the contributions of all participants to our present study.

## Conflict of interest

The authors declare that the research was conducted in the absence of any commercial or financial relationships that could be construed as a potential conflict of interest.

## Publisher’s note

All claims expressed in this article are solely those of the authors and do not necessarily represent those of their affiliated organizations, or those of the publisher, the editors and the reviewers. Any product that may be evaluated in this article, or claim that may be made by its manufacturer, is not guaranteed or endorsed by the publisher.
